# *TLR4* rs41426344 increases susceptibility of rheumatoid arthritis (RA) and juvenile idiopathic arthritis (JIA) in a central south Chinese Han population

**DOI:** 10.1186/s12969-017-0137-5

**Published:** 2017-02-21

**Authors:** Yan Wang, Lianghui Chen, Fang Li, Meihua Bao, Jie Zeng, Ju Xiang, Huaiqing Luo, Jianming Li, Liang Tang

**Affiliations:** 1 Department of Human Anatomy, Histology and Embryology, Institute of Neuroscience, Changsha Medical University, No. 1501 Lei Feng Road,, Wangcheng District, Changsha City, Hunan Province 410219 People’s Republic of China; 2 School of Basic Medical Science, Changsha Medical University, Changsha, People’s Republic of China; 3 Experiment center for Function, Changsha Medical University, Changsha, People’s Republic of China; 4 Department of Neurology, Xiang-ya Hospital, Central South University, Changsha City, Hunan Province China

**Keywords:** Genetic association, Toll like receptors 4 (*TLR4*), Rheumatoid arthritis (RA), Juvenile idiopathic arthritis (JIA), Chinese Han population

## Abstract

**Background:**

The aim of the study was to determine whether polymorphisms in toll-like receptor 4 (*TLR4*) confer susceptibility to rheumatoid arthritis (RA) and juvenile idiopathic arthritis (JIA) in a central south Chinese Han population.

**Methods:**

Genotyping for six well studied polymorphisms (rs4986790, rs4986791, rs10759932, rs41426344, rs11536889 and rs7873784) in *TLR4* gene were conducted in 1074 unrelated patients with RA and 1692 healthy control subjects, as well as in 217 unrelated patients with JIA and 378 healthy control subjects using direct sequencing technique. Comparisons between cases and controls in alleles, genotypes and haplotypes were carried out using Fisher’s exact test.

**Results:**

Significant genetic associations were detected between the 3’UTR rs41426344C and RA (*p* < 0.001, *p*_adj_ < 0.001, OR = 2.24) and JIA (*p* < 0.001, *p*_adj_ < 0.001, OR = 2.05). In addition, rs4986790G was found to be significantly associated with the susceptibility for RA (*p* = 0.005, *p*_adj_ = 0.03, OR = 3.43), but not for JIA (*p =* 0.06, *p*_adj_ = 0.36, OR = 2.65). Furthermore, significant increasing in the distributions of haplotypes H4 and H10 in RA (H4*: p* = 0.001, OR = 1.13; H10: *p* = 0.001, OR = 1.15) and JIA (H4*: p* = 0.04, OR = 2.06; H10: *p* = 0.02, OR = 2.47) were also found. Moreover, the frequency of rs41426344C significantly increased in RF-positive and anti-CCP positive subjects both in RA (RF^+^: *p* <0.0001, OR = 2.33; anti-CCP^+^: *p* =0.008, OR = 2.79) and JIA (RF^+^: *p* =0.02, OR = 2.91; anti-CCP^+^: *p* = 0.02, OR = 2.78).

**Conclusions:**

Our study suggested that rs41426344 and rs4986790 of *TLR4* might contribute to RA, and rs41426344 might contribute to JIA pathogenesis in central south Chinese Han population.

**Electronic supplementary material:**

The online version of this article (doi:10.1186/s12969-017-0137-5) contains supplementary material, which is available to authorized users.

## Background

Rheumatoid arthritis (RA) is an autoimmune disease characterized by progressive particular damage caused by inflammatory cells and synoviocytes and was thought to be caused by complex interaction of multiple susceptibility genes and environmental factors [1]. It affects approximately 0.32% Chinese Han population and 1% Caucasian respectively. Juvenile idiopathic arthritis (JIA) refers to a group of chronic childhood arthropathies of unknown aetiology [2]. Chronic arthritis is a common feature of RA and JIA. Familiar and twins studies have provided robust evidence for the role of genetic factors in these diseases [3, 4].

Toll-like receptors (TLRs) play important roles in the recognition of inflammatory diseases caused by invading microorganisms. They have been also increasingly suggested to have important roles in RA and JIA [5, 6]. There are 13 structurally unique members identified in TLRs family. Toll-like receptor 4 (TLR4), one of the important member of TLRs, plays a key role in the process of the innate immune response, and activates the nuclear factor-κB (NF-κB) signaling pathway by binding to lipopolysaccharide (LPS), which was identified to be an important mechanism in the development of rheumatic diseases [7–11].

The *TLR4* gene consisting of three exons is located on chromosome 9q32–33 [12]. Previous studies have reported that some polymorphisms in the *TLR4* coding/non-coding region, in particular Asp299Gly polymorphism, are associated with a blunted receptor activity and a subsequently diminished inflammatory response in humans [13–16]. Furthermore, variants in the *TLR4* were also reported to be associated with lymphoid tissue lymphoma [17], Hodgkin lymphoma [17], cancer [18] and ischemic cerebrovascular disease [19]. Surprisingly, relatively few genetic studies reported significant associations of polymorphisms in *TLR4* with RA and JIA susceptibility. Most studies have focused on the correlation between two well known *TLR4* polymorphisms (Asp299Gly and Thr399Ile) and RA and JIA, while inconclusive or contradictory results were observed [20, 21]. To our knowledge, only three studies with relatively small sample size have investigated the association between variants in the *TLR4* and RA in Chinese Han population [22–24], and negative result was also reported [23, 24]. In addition, no research conducted on the association between *TLR4* polymorphisms and JIA in central Chinese Han population was found. Thus, the role of *TLR4* in RA and JIA in central Chinese Han population remains unclear.

In present study, we aimed to examine the possible associations of *TLR4* polymorphisms with auto-antibody levels in RA and JIA susceptibility in a central south Chinese Han population.

## Methods

### Sample collection

The study was approved by the Ethical Committee at Changsha Medical University (EC/14/013, 06/11/2014). Written, informed consents for genetic analysis were obtained from all subjects or their guardians. A total of 1074 unrelated patients (Female/Male: 842/232; age: 41.7 ± 11.6 years) who met the American College of Rheumatology (The American Rheumatism Association) 1987 revised criteria for RA [25] and 217 unrelated patients (boy/girl: 178/39; age: 6.3 ± 3.1 years) who fulfilled the EULAR JIA criteria were recruited from the first affiliated hospital, Changsha Medical University. Rheumatoid factor (RF) and anti-cyclic citrullinated peptide (anti-CCP) status were determined for all the patients. The erythrocyte sedimentation rate (ESR) was tested by Westergren method. The auto-antibody levels were detected by Enzyme-linked immunosorbent assay (ELISA). In addition, 1692 unrelated control subjects without the history of RA and 378 unrelated control subjects without the history of JIA (matched for ethnicity, gender and age) for this study were also enrolled. The control subjects were healthy individuals who took the health examination in the first affiliated hospital, Changsha Medical University. All participants were Chinese Han population in origin.

### Genotyping

A combination of 6 well-studied informative *TLR4* SNPs (Two functional variants [rs4986790 (Asp299Gly) and rs4986791 (Thr399Ile) in exon 3, one variant (rs10759932) in 5’UTR and three variants (rs41426344, rs11536889 and rs7873784) in 3’UTR were genotyped in RA, JIA and healthy controls. Genomic DNA was extracted from peripheral leukocytes using the standard phenol–chloroform method [26]. The multiplex PCR was carried out on the ABI Veriti Thermal Cycler (Applied Biosystems, Foster City, CA). Genotyping was conducted using direct sequencing by the ABI 3730XL DNA Sequencer (Applied Biosystems, Foster City, CA). The PCR primers and sequencing probes were shown in Additional file 1: Table S1.

### Statistical analysis

Hardy-Weinberg equilibrium (HWE) was tested in the cases and controls using a classic chi-square test with 1° of freedom. The statistical analysis was performed using SHESIS (http://analysis.bio-x.cn/SHEsisMain.htm). Individual analyses of associations between *TLR4* polymorphisms and RA and JIA, as well as clinical features were performed by comparing alleles and genotypes in cases and controls using Fisher’s exact test. The corresponding ORs and 95% confidence intervals (CI) were assessed using a standard logistic regression analysis. Bonferroni correction was applied to adjust the *p* value (*P*_adj_) in multiple comparisons. Analysis of haplotype diversity was performed using the expectation-maximization algorithm (EM). Specific *P* values and ORs and 95% confidence intervals (CI) were obtained by comparing each haplotype with the more common haplotype in the population using Fisher’s exact test. Statistical significance was set at *p* < 0.05.

## Results

Clinical features such as erythrocyte sedimentation rate (ESR), C-reactive protein (CRP), IgA, IgG, IgM were shown in Table 1. For JIA, the patients can be classified into five subtypes (systemic JIA, polyarticular (RF^+^ and RF^−^) JIA, pauciarticular JIA, psoriatic JIA and other JIA). There were 21 (9.6%), 52 (23.7%) (RF^+^: 15 (6.9%); RF^−^: 37 (16.8%)), 111 (51.2%), 28 (12.9%) and 5 (2.3%) separately for each subtype.

**Table 1:** Clinical characteristics of RA and JIA patients and heathy controls

Clinical characteristics	RA (Mean ± SD)	Control (Mean ± SD)	*p*	JIA (Mean ± SD)	Control (Mean ± SD)	*p*
Sex ratio (Female/Male)	3.63 (842/232)	3.47 (1314/378)	0.17	4.56 (178/39)	4.72 (312/66)	0.89
Age (years)	41.7 ± 11.6	39.6 ± 13.2	0.75	6.3 ± 3.1	6.7 ± 2.5	0.75
Onset age, years	49.5 ± 7.9	–	–	7.5 ± 4.9	–	–
Bone erosions n (%)	421 (39.1%)	–	–	46 (21.3%)	–	–
Shared epitope	467 (43.5%)	–	–	79 (36.4%)	–	–
DAS28	4.7 ± 1.3	–	–	3.4 ± 1.1	–	–
ESR (mm/h) (0–10 mm/h)	33.2 ± 12.5	4.5 ± 2.2	<0.001	34.5 ± 19.3	4.3 ± 2.4	<0.001
CRP (mg/l) (0.8–8 mg/l)	25.8 ± 12.2	4.9 ± 1.3	<0.001	19.3 ± 37.5	3.77 ± 1.4	<0.001
IgA mg/mL (0.71–3.35 mg/mL)	12.2 ± 2.7	2.88 ± 1.4	<0.001	10.2 ± 2.4	2.59 ± 1.7	<0.001
IgG mg/mL (7.6–16.6 mg/mL)	45.7 ± 5.4	10.3 ± 4.1	<0.001	29.6 ± 5.1	9.7 ± 1.3	<0.001
IgM mg/mL (0.48–2.12 mg/mL)	6.6 ± 1.3	1.78 ± 2.1	<0.001	5.4 ± 1.7	1.88 ± 1.02	<0.001
RF^+^, %	826 (76.9%)	0 (0%)		37 (16.9%)	0 (0%)	
CCP^+^, %	766 (71.3%)	0 (0%)		33 (15.4%)	0 (0%)	

*Abbreviation*: *SD* Standard Deviation, *ESR* erythrocyte sedimentation rate, *CRP* C-reactive protein, *RF* rheumatoid factor, *JIA* juvenile idiopathic arthritis, *RA* Rheumatoid arthritis

Disease activity score 28(DAS28): a score for evaluation of RA activity by assessing the state of 28 joints; anti-CCP: anti-cyclic citrullinated peptide

### Single-locus association

All variants in cases and controls were in Hardy-Weinberg equilibrium (HWE) (*p* > 0.05). Genotype data for the 6 *TLR4* SNPs successfully typed in the central south Chinese Han population cases and controls were examined by single-marker analysis (Tables 2 and 3). Genotype analysis showed that the distribution of rs41426344 CC was significantly higher in RA and JIA patients compared with controls, even after the Bonferroni’s correction (RA: *p* < 0.001, *p*_adj_ < 0.001, OR [CI95%]: 3.75 [2.51–5.6]; JIA: *p* = 0.0002, *p*_adj_ = 0.0006, OR [CI95%]: 4.79 [1.97–11.67]). The frequencies of rs41426344C in RA and JIA were 0.21 and 0.25 separately. Significant associations between rs41426344C and RA and JIA were observed in further allelic analysis (RA: *p* < 0.001, *p*_adj_ < 0.001, OR [CI95%]: 2.24 [1.76–2.85]; JIA: *p* < 0.001, *p*_adj_ < 0.001, OR [CI95%]: 2.05 [1.52–2.77]).

**Table 2:** Allele distributions of *TLR4* gene polymorphisms in RA, JIA and healthy controls

			RA					JIA				
SNPs (MAF)	Region	Position	Case (freq.)	Control (freq.)	*P*	*P* ^*a*^_adj_	OR [95%CI]^b^	Case (freq.)	Control (freq.)	*P*	*P* ^*a*^_adj_	OR [95%CI]^b^
rs10759932(C)	5’UTR	27786349	0.32	0.29	0.37	–	1.10 [0.89–1.34]	0.26	0.26	0.86	–	1.03 [0.78–1.34]
rs4986790 (G)	Exon 3	27796507	0.02	0.006	**0.005**	**0.03**	**3.43 [1.39–8.45]**	0.02	0.008	0.06	–	2.65 [0.93–7.49]
rs4986791 (T)	Exon 3	27796807	0.05	0.04	0.03	0.18	1.75 [1.09–2.82]	0.07	0.05	0.12	–	1.46 [0.90–2.37]
rs41426344 (C)	3’UTR	27799138	0.21	0.13	**<0.001**	**<0.001**	**2.24 [1.76–2.85]**	0.25	0.14	**<0.001**	**<0.001**	**2.05 [1.52–2.77]**
rs11536889 (C)	3’UTR	27799336	0.22	0.19	0.07	–	1.24 [0.98–1.56]	0.23	0.19	0.19	–	1.21 [0.91–1.61]
rs7873784 (C)	3’UTR	27800141	0.15	0.12	0.03	0.18	1.35 [1.03–1.77]	0.13	0.14	0.68	–	0.93 [0.66–1.31]

*Abbreviation*: *SNP*, single nucleotide polymorphism, *MAF* minor allele frequency, *OR* odds ratio, *95% CI* 95% confidence intervals, not calculated, *RA* Rheumatoid arthritis, *JIA* juvenile idiopathic arthritis, *Freq* frequency; p_adj_, p_adjusted_

^a^The Bonferroni’s correction was carried out to adjust the *p* value

^b^OR and 95% CI are calculated for the minor allele of each SNP

**Table 3:** Distribution of the genotypes of *TLR4* gene polymorphisms in RA and JIA cases and controls

SNPs	Genotype	Control no.	RA no.	OR [95%CI]	*p* ^a^	*p* ^b^_adj_	Control no.	JIA no.	OR [95%CI]	*p* ^a^	*p* ^b^_adj_
rs10759932 (C)	TT	844	498	0.87 [0.74–1.01]	0.07	–	204	125	1.16 [0.83–1.62]	0.39	–
	CT	696	466	1.09 [0.94–1.28]	0.24	–	151	73	0.76 [0.54–1.08]	0.13	–
	CC	143	108	1.21 [0.93–1.57]	0.15	–	23	19	1.48 [0.79–2.79]	0.22	–
rs4986790 (G)	AA	1671	1029	**0.28 [0.17–0.49]**	**<0.001**	**<0.001**	372	208	0.37 [0.13–1.06]	0.06	–
	GA	21	45	**3.47 [2.06–5.87]**	**<0.001**	**<0.001**	6	9	2.68 [0.94–7.64]	0.06	–
	GG	0	0	–	–	–	0	0	–	–	–
rs4986791 (T)	CC	1590	986	0.72 [0.53–0.96]	0. 03	0.09	399	186	0.58 [0.35–0.97]	0.04	0.12
	CT	100	85	1.37 [1.01–1.84]	0.04	0.12	39	30	1.65 [0.99–2.73]	0.05	–
	TT	2	3	–	–	–	0	1	–	–	–
rs41426344 (C)	GG	1287	615	**0.42 [0.36–0.49]**	**<0.001**	**<0.001**	280	127	**0.49 [0.35–0.70]**	**<0.001**	**<0.001**
	GC	369	378	**1.94 [1.64–2.31]**	**<0.001**	**<0.001**	91	72	1.57 [1.08–2.26]	0.02	0.06
	CC	36	81	**3.75 [2.51–5.6]**	**<0.001**	**<0.001**	7	18	**4.79 [1.97–11.67]**	**0.0002**	**0.0006**
rs11536889 (C)	GG	1113	647	**0.79 [0.67–0.92]**	**0.003**	**0.009**	242	133	0.89 [0.63–1.26]	0.51	–
	GC	518	372	1.20 [1.02–1.41]	0.03	0.09	125	70	0.96 [0.67–1.37]	0.84	–
	CC	60	54	1.44 [0.99–2.09]	0.06	–	11	14	2.3 [1.02–5.16]	0.04	0.12
rs7873784 (C)	GG	1308	790	0.81 [0.68–0.97]	0.02	0.06	278	165	1.14 [0.78–1.68]	0.50	–
	GC	358	268	1.24 [1.03–1.48]	0.02	0.06	96	48	0.83 [0.56–1.23]	0.37	–
	CC	25	16	1.01 [0.54–1.89]	1.00	–	4	4	1.76 [0.43–7.09]	–	–

*Abbreviation*: *SNP* single nucleotide polymorphism, *OR* odds ratio, *95% CI* 95% confidence intervals; −, not calculated, *RA* Rheumatoid arthritis, *JIA* juvenile idiopathic arthritis

^a^ *P* value were calculated using Fisher’s exact test

^b^The Bonferroni’s correction was carried out to adjust the *P* value

The distribution of the rs4986790GA in RA cases was significantly higher than that in controls (*p* < 0.001, *p*_adj_ < 0.001, OR [CI95%]: 3.47 [2.06–5.87]). And allelic analysis of the RA cohort revealed that the frequency of the rs4986790G was significantly higher in patients (2%) compared with controls (0.06%) with an OR equal to 3.43 (*p* = 0.005, *p*_adj_ = 0.03, OR [CI95%]: 3.43 [1.39–8.45]), which indicated that G allele in rs4986790 might reveal a strong risk factor for RA in central south Chinese Han population.

No association was detected between other SNPs in the 3’UTR (rs11536889 and rs7873784) and 5’UTR (rs10759932) of the *TLR4* gene and RA and JIA (*p* > 0.05). And no notable association was detected between both genotypes and alleles in rs4986790 and JIA (*p* > 0.05).

### Haplotype analysis

Haplotypes were predicted for 6 SNPs using PLINK 1.09 (http://pngu.mgh.harvard.edu/~purcell/plink/). Ten haplotypes in RA and JIA separately with a frequency > 1% were predicted in both cases and controls accounting for > 90% of all the haplotypes. The haplotype 1(H1) (TACGGG) containing rs10759932T, rs4986790A, rs4986791C, rs41426344G, rs11536889G, rs7873784G was the most common haplotype with a frequency of approximately 43% in RA and 42% in JIA. However, no association was found between H1 and RA and JIA (*p* > 0.05). Additionally, we observed a marginally significant increase in the distribution of H4 (TGTCCG) and H10 (CGTCCG) in RA compared with that in the controls (H4*: p* = 0.001, OR [95%CI] = 1.13 [0.77–1.26]; H10: *p* = 0.001, OR [95%CI] = 1.15 [1.02–1.56]) (Table 4). Similar results were found in H4 and H10 in JIA and controls (TGTCCG*: p* = 0.04, OR [95%CI] = 2.06[1.01–4.21]; H10: *p* = 0.02, OR [95%CI] = 2.47[1.11–5.49]) (Table 4).

**Table 4:** Haplotype analysis of RA and JIA cases and the healthy controls in the *TLR4* genes

NO.	haplotype^a^	RA		OR [95CI%], *P* ^*b*^	JIA		OR [95CI%], *P* ^*b*^
		Control (freq.)	Case (freq.)		Control (freq.)	Case (freq.)	
H1	T A C G G G	0.47	0.43	1.03 [0.99–1.17],0.29	0.45	0.42	0.72 [0.52–1.01],0.06
H2	T A C G G C	0.06	0.12	1.07 [0.83–1.33],0.07	0.06	0.10	1.74 [0.95–3.21],0.07
H3	T A C G C G	0.11	0.09	0.54 [0.22–1.11],0.13	0.10	0.07	0.66 [0.36–1.23],0.19
H4	T G T C C G	0.02	0.08	**1.13 [0.77–1.26],0.001**	0.04	0.08	**2.06 [1.01–4.21] 0.04**
H5	T A C C C C	0.003	0.01	1.09 [0.76–1.45],0.06	0.003	0.006	1.75 [0.11–2.86],0.39
H6	C A C G G G	0.06	0.06	1.01 [0.99–1.03],0.89	0.04	0.06	1.54 [0.72–3.31],0.26
H7	C A C G C G	0.01	0.02	1.10 [0.98–1.24],0.57	0.02	0.02	0.87 [0.26–2.93],0.78
H8	C A C G C C	0.003	0.001	0.86 [0.63–1.05],0.69	0.002	0.003	1.74 [0.11–2.04],0.54
H9	C A C C G G	0.14	0.11	0.98 [0.97–0.99],0.07	0.15	0.11	0.70 [0.42–1.16],0.17
H10	C G T C C G	0.03	0.13	**1.15 [1.02–1.56],0.001**	0.03	0.07	**2.47 [1.11–5.49],0.02**

*Abbreviation*: *SNP* single nucleotide polymorphism, *OR* odds ratio, *95% CI* 95% confidence intervals; −, not calculated, *RA* Rheumatoid arthritis, *JIA* juvenile idiopathic arthritis, *Freq* frequency

^a^Haplotype structure of *TLR4* for RA and JIA were rs4986790, rs4986791, rs10759932,rs41426344, rs11536889, rs7873784

^b^ *P* value were calculated using Fisher’s exact test

### Allelic/Genotypic distribution of RF and anti-CCP in RA and JIA

Data were available on autoantibody levels including information on circulating RF and anti-CCP. Carriage of rs41426344C significantly increased in RF-positive (RF^+^ vs. RF^−^ : 0.17 vs. 0.08) and anti-CCP positive (anti-CCP^+^ vs. anti-CCP^−^ : 0.15 vs. 0.06) subjects in RA (RF^+^: *p* <0.0001, OR [95%CI] = 2.33 [1.65–3.01]; anti-CCP^+^: *p* =0.008, OR [95%CI] = 2.79[1.28–6.11]) and JIA (RF^+^ vs. RF^−^ : 0.19 vs. 0.08; anti-CCP^+^ vs. anti-CCP^−^ : 0.16 vs. 0.05) (RF^+^: *p* =0.02, OR [95%CI] = 2.91 [1.11–7.56]; anti-CCP^+^: *p* =0.02, OR [95%CI] = 2.78 [1.21–6.74]) (Table 5). Allele and genotype frequencies were not different after stratification by anti-CCP status for rs4986790 that was shown to be associated with RA and JIA in our study (Table 5).

**Table 5:** Rs4986790 and rs41426344 allele/genotype frequencies and autoantibody levels in patients with RA and JIA

SNPs	Allele/Genotypes	RA		*P* ^*a*^,OR [95%CI]	JIA		*P* ^*a*^,OR [95%CI]	RA		*P* ^*a*^,OR [95%CI]	JIA		*P* ^*a*^,OR [95%CI]
		RF^+^ (freq.)	RF^−^ (freq.)		RF^+^(freq.)	RF^−^ (freq.)		Anti-CCP^+^(freq.)	Anti-CCP^−^(freq.)		Anti-CCP^+^(freq.)	Anti-CCP^−^(freq.)	
rs4986790	G	0.11	0.13		0.15	0.14		0.25	0.21		0.13	0.11	
	A	0.89	0.87	0.74,0.94 [0.65–1.36]	0.85	0.86	0.82,1.07 [0.59–1.97]	0.75	0.79	0.55,1.19 [0.66–2.15]	0.87	0.89	0.84,1.26 [0.97–1.93]
	GG	0.0	0.0	–	–	–	–	0.0	0.0	–	0.0	0.0	–
	GA	0.33	0.29	0.51,1.17 [0.73–1.86]	0.31	0.30	0.84,1.06 [0.57–1.99]	0.34	0.32	0.75,0.88 [0.42–1.86]	0.29	0.27	0.64,1.22 [0.79–1.88]
	AA	0.67	0.71	0.92,0.98 [0.63–1.52]	0.69	0.70	0.84,0.94 [0.50–1.76]	0.66	0.68	0.84,0.93 [0.45–1.90]	0.71	0.73	0.79,0.46 [0.22–0.97]
rs41426344	C	0.17	0.08		0.19	0.08		0.15	0.06		0.16	0.05	
	G	0.83	0.92	**<.0001,2.33 [1.65–3..01]**	0.82	0.92	**0.02,2.91 [1.11–7.56]**	0.85	0.94	**0.008,2.79 [1.28–6.11]**	0.84	0.95	**0.02,2.78 [1.21–6.74]**
	CC	0.04	0.01	**0.01,1.45 [0.48–4.26]**	0.04	0.015	**0.001,3.23 [0.39–26.79]**	0.03	0.01	–,2.34 [0.27–20.45]	0.02	0.01	–,2.45 [0.36–18.75]
	CG	0.30	0.13	**<.0001,2.82 [1.90–4.19]**	0.30	0.13	**0.009,2.76 [1.26–6.05]**	0.23	0.09	**0.003,3.37 [1.48–9.43]**	0.22	0.10	**0.002,2.94 [1.39–8.25]**
	GG	0.66	0.86	**<.0001,0.37 [0.25–0.53]**	0.65	0.85	**0.002,0.32 [0.15–0.69]**	0.74	0.90	**<.0001,0.21 [0.09–0.48]**	0.76	0.89	**<.0001,0.34 [0.11–0.85]**

*Abbreviation*: *SNP* single nucleotide polymorphism, *OR* odds ratio, *95% CI* 95% confidence intervals; −, not calculated, *RF* rheumatoid factor, *anti-CCP* anti-cyclic citrullinated peptide

^a^ *P* value were calculated using Fisher’s exact test

## Discussion

In the current study, 1074 RA, 217 JIA and 2070 healthy controls were genotyped for six polymorphisms in the *TLR4* gene that was previously reported to be associated with autoimmune diseases. The data showed that the frequencies of *TLR4* rs4986790G in RA cases, as well as rs41426344C in JIA cases significantly increased than that in the controls, which was, to our knowledge, the first study to demonstrate associations between the two common polymorphisms and RA and JIA in central Chinese Han population using case-control design.

TLRs play important roles in both innate and adaptive immune responses that invading by microorganisms [27]. The chronic inflammation and the well-recognized interactions of TLRs with numerous endogenous ligands have implicated this pathway in a number of disease states including RA and JIA [27, 28]. As a member of TLRs, TLR4 has been considered to recognize not only the LPS component of gram-negative bacteria but also the mouse mammary tumor virus [29, 30]. In particular, TLR4 has been identified as an important part of investigation in understanding arthritides pathogenesis. It has been also demonstrated that TLR4 is over-expressed in RA synovium [31]. Investigations using animal models of inflammatory arthritis also implicate TLR4 in RA. Mice with non-functional TLR4 or mice deficient of MyD88 are protected from inflammatory arthritis [32]. As for JIA, Donn R et al. indicated that the macrophage migration inhibitory factor (MIF) have been reported to be associated with JIA [33]. And a relationship between MIF and TLR4 was found in a study of MIF-deficient mice [34], which supported the hypothesis that TLR4 is a risk factor for investigation in JIA.

The *TLR4* Asp299Gly (rs4986790) is a functional allele located in the exon 3 region of *TLR4* gene and was known to cause an aspartic acid to glycine replacement, which alter its extracellular domain and potentially modify its binding affinity. The strong association between *TLR4* Asp299Gly polymorphism and RA disease susceptibility has been reported in a Dutch cohort [35], but not in Irish, British and Spanish populations [35–37]. And no positive results was found between *TLR4* Asp299Gly and JIA in UK Caucasian and Indian [5, 38]. In our study, the frequency of Asp299Gly polymorphism in central south Chinese Han population was higher than that in other Chinese Han population populations [23, 39, 40], but was similar with that in Caucasian populations [41–44]. And a significant association was detected between *TLR4* Asp299Gly and RA in central south Chinese Han population compared to healthy controls. To our knowledge, this is the first study that a significant association between *TLR4* Asp299Gly and RA in Chinese Han population was reported. Interestingly, negative result was shown by Zheng [23] and Yuan [24]. The complex genetic ethnic specificity in Chinese Han populations might contribute to the difference.

Notable, the rs41426344 appeared to be significantly associated with both RA and JIA in central south Chinese Han population. Both rs41426344C allele and CC genotype are increased in RA cases, which was similar with the result reported by Zheng [22]. There were already evidences suggesting that the rs41426344 may act as susceptibility loci with diseases [44]. Cheng et al. suggested that rs41426344 may be a functional site, which could attenuate the LPS-induced transmembrane signaling through the alteration of post-transcriptional regulation of 3’UTR and target gene expression [41]. In addition, no significant association was found between other two 3’UTR SNPs (rs11536889 and rs7873784) and RA and JIA. Though the absence of association in these two loci was detected in our present study, we cannot exclude the possible effect of these two SNPs on RA and JIA development in other populations for genetic polymorphisms often vary between ethnic groups. Thus, replication in other populations is needed before these results can be generalized.

## Conclusions

We observed significant associations between RA and JIA disease susceptibility and a *TLR4* variant (rs41426344) in a set of RA and JIA patients, as well as rs4986790 in RA patients and healthy individuals in central south Chinese Han population. Our finding needs to be confirmed in other larger numbers of Chinese Han population cohorts. And to identify the potential mechanisms by which variant in rs41426344 and rs4986790 affects *TLR4* and RA and JIA is necessary.

## Additional file

Additional file 1: Table S1. The PCR and sequencing primers of *TLR4* SNPs. (DOC 30 kb)
