# Supplementary material for: TLR4 rs41426344 increases susceptibility of rheumatoid arthritis (RA) and juvenile idiopathic arthritis (JIA) in a central south Chinese Han population
Source: Pediatr Rheumatol Online J. 2017 Feb 21;15:12. doi: 10.1186/s12969-017-0137-5 (PMC5320652; doi:10.1186/s12969-017-0137-5)
Supplement: Additional file 1: Table S1. — The PCR and sequencing primers of TLR4 SNPs. (DOC 30 kb) [file 12969_2017_137_MOESM1_ESM.doc]

Table 1s. The PCR and sequencing primers of *TLR4* SNPs.

| SNP | PCR primers |  | Sequencing primer(5'→3') |
| --- | --- | --- | --- |
|  | 5'→3' | 5'→3' |  |
| rs10759932 | ATTTTACAGACCAGAAAGTAATAACTAG | ATTGCTGGATCATGTGGTAAAT | ATTTGACTACCATTGCGTATCTT |
| rs4986790 | TTAGAAATGAAGGAAACTTGGAAAAG | TTTGTCAAACAATTAAATAAGTGATTAATA | CCCCATCCAGAGTTTAGCCCT |
| rs4986791 | TCTCAAAGTGATTTTGGGTTAA | GTTTCAAATTGGAATGCTGGA | TTAGAAATGAAGGAAACTTGGAAAAG |
| rs41426344 | TGGTCTTATCATGGACAATTTG | GAAAAAGATAAACTTCTGCTGGTA | GTCATTCCAAAGTTATTGCCTA |
| rs11536889 | CTCCTTGACCACATTTTGTTAA | AGGCTGGATGAACATTCTTTT | ATTTCCGCTTCCTGGTCTTAT |
| rs7873784 | TGAGGAAATAGGGAGTTGTCTAAT | GTGCATAATACAGTATTGTTCATTGTA | AAAAGGAGGAAGGGAGAAATG |
